# Fiber-free white flour with fructose offers a better model of metabolic syndrome

**DOI:** 10.1186/1476-511X-12-44

**Published:** 2013-03-28

**Authors:** Faridah Amin, Anwar H Gilani

**Affiliations:** 1 Natural Products Research Division, Department of Biological and Biomedical Sciences, Aga Khan University Medical College, Karachi 74800, Pakistan; 2 College of Health Sciences, Mekelle University, Mekelle, Ethiopia

**Keywords:** Metabolic syndrome, Fructose-fed rats, Whole wheat flour, Refined/white flour, Animal model

## Abstract

**Background:**

The metabolic syndrome (MS) is a combination of metabolic abnormalities that lead to an increased risk of cardiovascular diseases. Due to its rising incidence and demanding life-long use of multiple drugs, there is a growing interest in testing and developing new allopathic, complementary and alternative therapies for controlling or curing disorders of MS. The discovery of new therapeutic modalities requires animal models of disease and currently available models have limitations. Developing an appropriate animal model for MS to achieve various therapeutic targets remains a challenge and this study aims to develop a rat model which closely depicts MS in humans.

**Methodology:**

Rat model of MS was developed by replacing 60% of diet with fructose. Four groups of Sprague–Dawley rats were either given whole wheat or refined flour with and without fructose for 8 weeks. Data were analyzed on SPSS and Graphpad Prism using ANOVA with Tukey’s and Bonferonni tests for multiple group comparison. A p-value of less than 0.05 was considered significant for differences between groups.

**Results:**

Replacing whole wheat with refined wheat flour in rat chow in 60% fructose-fed Sprague–Dawley rats resulted in hypertension (p 0.01), hyper-insulinemia (p < 0.001), hyperglycemia (p 0.03) and a reduction in HDL levels (p 0.002) at 4 weeks while hyper-triglyceridemia (p 0.001) with endothelial dysfunction was observed at 8 weeks.

**Conclusion:**

It is concluded that the refined wheat flour with 60% fructose in diet hastens the development of metabolic syndrome in 4 weeks and replacing whole wheat flour with refined flour in diet induces a more effective abnormality including a low HDL. Further studies may be directed to assess the associated pathological changes, which can be used to study the effect of different therapeutic modalities on an animal model of MS with low HDL.

## Background

The metabolic syndrome (MS) is a combination of certain metabolic abnormalities such as obesity, insulin resistance, diabetes, dyslipidemia and hypertension, which in combination are associated with a risk of coronary heart disease, stroke, and cardiovascular mortality greater than that of each abnormality [1].

Over the past two decades, the incidence of MS is rising. According to the Centers for Disease Control and Prevention, approximately 34% of American adults meet the criteria for MS [2]. Up to one-fourth of the European and South Asian adults have MS [3].

Despite the rising incidence, there is limited success to treat MS with available chemical drugs; hence more studies are warranted in the search of more efficacious and safer remedies.

Diet is a major factor in metabolic syndrome [4]. It is generally recommended that patients with MS should consume a diet with less saturated fat; increased vegetables, legumes, fruit and food with a low glycemic index and high fiber content [5]. There is also an ample evidence that consumption of more refined carbohydrates than cereal and whole grains is associated with a higher incidence of Diabetes mellitus [6], dyslipidemia [7], MS [8] and coronary heart disease (CHD) [9]. Similarly, whole grains which include wheat flour bran have not only been shown to increase the bioavailability of essential minerals in rats [10] but also have a favorable effect on diabetic patients [11,12], metabolic markers [13], CHD [14], ischemic stroke [15] and total mortality [16].

In animal studies, whole grain wheat flour has been shown to produce metabolic changes, some of which may protect the organism against oxidative stress [17]. Although a study done two decades back showed favorable effects of white flour as compared to whole wheat flour on cholesterol levels in rats [18] but abundant literature on humans has clarified the fact that consuming white flour is associated with dyslipidemia [19].

Drug discovery and development depends on using animal models of disease before drugs are allowed for clinical trials. Besides genetic models of MS, such as the obese Zucker rat, MS is induced in rats through various protocols, which include administration of sucrose or fructose in diet. Various studies have shown induction of features of MS including hyper-triglyceridemia, hyper-insulinemia, hypertension and hyperglycemia when Wistar or Sprague–Dawley rats were given 60-66% fructose for 4–8 weeks [20-22]. A study comparing different protocols using fructose for induction of MS found that 60% fructose diet is a more effective way for induction of MS in rats than drinking 10% fructose solution [23]. In these studies, standard rat chow was used which includes whole wheat flour as a major component. Inducing MS by using existing animal models has limitations also because it does not induce an abnormality in HDL, which is an essential component of MS.

Refined or white flour as compared to whole wheat flour, known to increase postprandial glycemia, is hypothesized to induce signs of metabolic syndrome in rats more effectively and in a shorter period of time. Therefore, this study aims to compare the effects of replacing refined wheat flour with whole wheat flour to diet, in development of metabolic syndrome and to see, if it also induces endothelial dysfunction in fructose fed rats. Although there are ample data on this in human subjects, but to the best of our knowledge the effect of whole wheat flour and white flour in induction of MS in rats has not been compared previously especially the effect of refined flour in reducing the HDL levels.

## Results

The weight gain, fluid and diet intake were similar among groups at 8 weeks. All other variables were not significantly different between groups at baseline (Table 1).

**Table 1:** Comparison of baseline parameters of metabolic syndrome among different groups

**Parameters of metabolic syndrome**	**Whole wheat flour diet**	**Refined wheat flour diet**	**Fructose and whole wheat flour diet**	**Fructose and fefined wheat flour diet**
**BP (mmHg)**	133 ± 2.86	125 ± 5.9	129 ± 2.23	124 ± 4.49
**FBS (mg/dl)**	112 ± 5.9	105 ± 6.63	108 ± 5.1	107 ± 9.25
**Chol (mg/dl)**	68 ± 4.9	58 ± 7.5	60 ± 5.17	58 ± 5.08
**TGL (mg/dl)**	37 ± 2.38	41 ± 1.8	48 ± 3.78	35 ± 1.54
**LDL (mg/dl)**	11 ± 1.24	14 ± 1.77	9 ± 0.88	14 ± 2.85
**HDL (mg/dl)**	58 ± 3.8	65 ± 3.44	54 ± 3.59	66 ± 4.64
**CRP (mg/dl)**	0.17 ± 0.007	0.12 ± 0.007	0.13 ± 0.008	0.16 ± 0.01
**Insulin (ng/dl)**	2.5 ± 0.34	2.7 ± 0.59	1.9 ± 0.46	1.4 ± 0.36

Blood pressure (BP), Fasting blood sugar (FBS), Total cholesterol (Chol), Triglycerides (TGL), Low density lipoproteins (LDL), High density lipoproteins (HDL), C- reactive protein (CRP) and serum insulin expressed as Means ± SEM.

No significant difference was found between groups at p of 0.05.

At 4 weeks (Table 2), the BP of whole wheat flour with fructose (p 0.01) and refined wheat flour with fructose group was significantly higher than their respective controls (p 0.006). There was no change in serum cholesterol, TGL, LDL and CRP between groups (p > 0.05). FBS (p 0.005) while serum insulin level (p < 0.001) was significantly higher in refined wheat flour with fructose group. Interestingly, the HDL was significantly lower in refined wheat flour with fructose as compared to whole wheat flour with fructose-fed group (p 0.014).

**Table 2:** Effect of different diets on parameters of metabolic syndrome at 4 weeks

**Parameters of metabolic syndrome**	**Whole wheat flour diet**	**Refined wheat flour diet**	**Fructose and whole wheat flour diet**	**Fructose and refined wheat flour diet**
**BP (mmHg)**	136 ± 3.78^ab^	125 ± 2.65^ab^	154 ± 3.78^c^	145 ± 4.54^ac^
**FBS (mg/dl)**	95 ± 8.02^a^	100 ± 12.05^a^	121 ± 10.9^ab^	156 ± 10.1^b^
**Chol (mg/dl)**	69 ± 5.28^a^	69 ± 5.97^a^	67 ± 3.98^a^	60 ± 2.57^a^
**TGL (mg/dl)**	38 ± 2.40^a^	36 ± 3.87^a^	38 ± 2.17^a^	41 ± 3.97^a^
**LDL (mg/dl)**	13 ± 1.77^a^	12 ± 1.91^a^	16 ± 1.69^a^	12 ± 1.52^a^
**HDL (mg/dl)**	54 ± 4.89^a^	48 ± 4.57^a^	60 ± 3.2^ab^	40 ± 3.39^ac^
**CRP (mg/dl)**	0.17 ± 0.018^a^	0.20 ± 0.014^a^	0.14 ± 0.01^a^	0.15 ± 0.01^a^
**Insulin (ng/dl)**	3.2 ± 0.54^a^	4.9 ± 0.84^a^	2.6 ± 0.49^a^	14.8 ± 1.87^b^

Blood pressure (BP), Fasting blood sugar (FBS), Total cholesterol (Chol), Triglycerides (TGL), Low density lipoproteins (LDL), High density lipoproteins (HDL), C-reactive protein (CRP) and serum insulin expressed as Means ± SEM.

^abc ^Within rows, between control and treated animals, means with different superscript letters differ significantly (p < 0.05).

At 8 weeks (Table 3), the BP remained high only in the fructose-fed refined wheat flour group compared to control (p 0.008). The FBS and serum insulin levels remained raised in fructose-fed refined flour group (p < 0.001), whereas, the whole wheat flour with fructose group although had a raised FBS level than its control (p 0.02), failed to show a difference in insulin levels (p > 0.05). The cholesterol levels did not differ significantly between groups, although the variable as a whole was significant (p 0.049). Due to a significantly low HDL in refined wheat flour with fructose and without fructose (p <0.001), as compared to whole wheat flour groups, the total cholesterol/HDL ratio was significantly raised in refined flour-fed with (p 0.02) and without fructose groups (p 0.002). The triglycerides level at 8 weeks was also significantly higher in refined flour fructose-fed rats than in control group (p < 0.001).

**Table 3:** Effect of different diets on parameters of metabolic syndrome at 8 weeks

**Parameters of metabolic syndrome**	**Whole wheat flour diet**	**Refined wheat flour diet**	**Fructose and whole wheat flour diet**	**Fructose and refined wheat flour diet**
**BP (mmHg)**	133 ± 4.63^a^	134 ± 4.72^a^	149 ± 6.98^ab^	162 ± 5.4^b^
**FBS (mg/dl)**	111 ± 5.37^a^	101 ± 9.0^a^	149 ± 7.29^b^	159 ± 11.24^b^
**Chol (mg/dl)**	67 ± 2.22^a^	51 ± 7.24^a^	68 ± 4.64^a^	68 ± 2.74^a^
**TGL (mg/dl)**	46 ± 4.32^ab^	47 ± 3.15^a^	64 ± 11.7^ab^	65 ± 2.27^b^
**LDL (mg/dl)**	13 ± 1.60^a^	20 ± 1.68^b^	17 ± 1.58^ab^	22 ± 1.62^b^
**HDL (mg/dl)**	57 ± 3.19^a^	24 ± 1.64^ab^	48 ± 4.1^a^	26 ± 2.85^ab^
**CRP (mg/dl)**	0.15 ± 0.01^a^	0.24 ± 0.03^a^	0.22 ± 0.02^a^	0.23 ± 0.02^a^
**Insulin (ng/dl)**	3.6 ± 0.54^a^	7.13 ± 0.76^a^	7.3 ± 1.45^a^	16.0 ± 1.86^b^

Blood pressure (BP), Fasting blood sugar (FBS), Total cholesterol (Chol), Triglycerides (TGL), Low density lipoproteins (LDL), High density lipoproteins (HDL), C-reactive protein (CRP) and serum insulin expressed as Means ± SEM.

^ab ^Within rows, between control and treated animals, means with different superscript letters differ significantly (p < 0.05).

**Figure 1:**
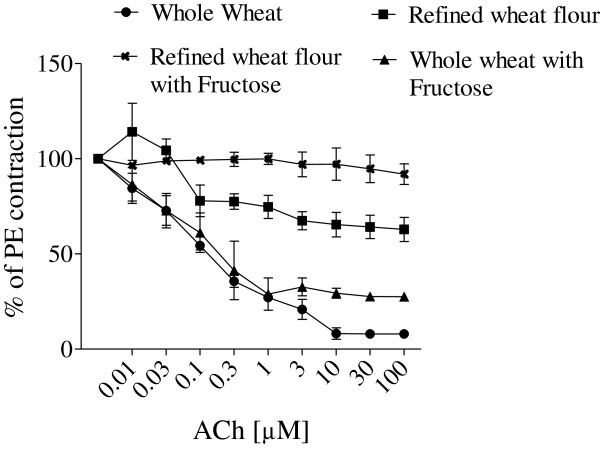
**Comparative effect of different diets on endothelial function. **Comparative effect of different diets on endothelial function measured in terms of alteration in the Acetylcholine (ACh) induced vasodilator effect constructed against phenylephrine (PE, 1 μM) induced sustained vascular contraction in rats. A dose dependent significant difference was seen between refined wheat with fructose and whole wheat with fructose groups as the ACh dose was increased from 0.03- 100 μM.

Figure 1 shows the dose-dependent inhibition of PE (1 μM)-induced endothelial resistance with ACh in percentage after a pre-contracted vascular preparation with intact endothelium. At ACh doses of 0.03 μM and above, fructose-fed groups with refined wheat and whole wheat flour were significantly different from each other (p < 0.001). At ACh doses of 0.3- 100 μM, all the groups were significantly different from each other (p < 0.001) except the whole wheat flour and whole wheat flour with fructose group, which did not show any difference up to ACh dose of 3 μM.

## Discussion

The results of our study show a hastened development of metabolic syndrome at 4 weeks with the use of refined wheat flour with 60% fructose in diet. High fasting serum insulin levels and a raised blood pressure developed and persisted at 4 weeks and 8 weeks respectively, whereas cholesterol/HDL ratio and hyper-triglyceridemia were significantly raised at 8 weeks. More importantly, there was evidence of endothelial dysfunction especially in groups treated with refined flour when the rats were sacrificed at 8 weeks. These results highlight how the use of refined wheat flour rather than whole wheat flour can negatively alter the metabolic profile in Sprague–Dawley rats in a few weeks. This may be due to a protective effect of whole wheat against oxidative stress, as seen in the past [17,24]. It is also hypothesized that the beneficial effects of whole grain flour are mediated by an improved insulin sensitivity, which can be partially explained by the lower glycemic indices of whole wheat flour in comparison to its fiber-depleted analog which is refined flour [25].

The weight, diet and fluid intake was similar among the groups, which is consistent with other studies reporting that a 60% fructose without a high fat diet does not lead to weight gain or adiposity as compared to controls [26,27]. It is also known from previous studies that obesity or adipocyte hypertrophy is not the only cause of insulin resistance/hyper-insulinemia [4]. Studies have shown that a high fat and high sugar diet can lead to insulin resistance, while defects in the insulin-signaling pathway have been reported with a high fat diet [28]. There is also report showing that out of the two aspects of a high fat and sugar diet, the refined sugar causes greater toxicity than the high fat content during glucose tolerance tests [29]. Besides, epidemiological studies revealed that increased consumption of whole grains due to its fiber content is associated with lower fasting insulin concentrations and glycaemic response [11].

It is known that high fructose feeding to rats is associated with oxidative stress and inflammatory response demonstrated by an increase in reactive oxygen species production by polymorphonuclear leukocytes and tissues and an increased plasma levels of IL1 and IL6 [30,31]. C-reactive protein (CRP) is an acute-phase protein secreted by the liver and it is used as a marker for subclinical inflammation and CVD risk but the link between CRP and insulin resistance remains unclear [24]. It is not clear whether dietary fructose alone can lead to a raised CRP in rats but dietary fiber intake is shown to be inversely correlated with serum CRP concentration [32]. In our study, although there was no significant difference between CRP levels among groups (p > 0.05) but CRP was found to be lower in whole wheat flour-fed group at 8 weeks, although the difference was insignificant (p > 0.05).

Endothelial dysfunction is also a pro-inflammatory state [33] and endothelium-dependent relaxation has been shown to be depressed in fructose-fed animal models with hypertension [34,35]. Our study showed similar results with enhanced endotheliopathy in rats, which were fed with combination of fructose and refined flour. Moreover, the rats fed on refined wheat flour with or without fructose had none or less dose-dependent endothelial relaxation respectively than rats which were fed whole wheat flour. In humans, whole grain consumption is known to reduce BP [36]. Similarly, in our study, the protection conferred by whole wheat on blood pressure at 8 weeks may be due to the high magnesium content in whole wheat flour, which is known to possess a calcium antagonist activity [25], in addition to its fiber content, which also has a role in reduction of blood pressure [37].

More importantly, previous studies have proposed consumption of a high-carbohydrate, high-fat diet by normal rodents to be an adequate rodent model to mimic the human metabolic syndrome and for testing potential therapeutic interventions [38]. These studies have shown that administration of fructose in diet with regular rat chow, which has whole wheat as a major constituent, leads to an increase in HDL along with other lipids [23,39]. Even the Zucker rats, which is a genetic model of metabolic syndrome has been shown to have an increased HDL, when fed with a high fat diet [40], which is a major limitation in the development of dyslipidemia. HDL is the predominant lipoprotein in rats [41,42], thus, it is not worth exploring changes in LDL levels in rats with different diets or therapeutic agents.

Fructose with whole wheat chow did not lead to a reduction in HDL level probably because whole wheat flour is already known to have a protective effect on cholesterols without lowering HDL [43]. Moreover, among healthy people, a high refined-carbohydrate diet has been shown to reduce HDL [7]. Keeping in view the fact that a low HDL level is an essential component of metabolic syndrome in humans [44] and insulin resistance is strongly associated with a low HDL level [45], our attempt was to develop a model ensuring an abnormality in HDL levels also.

In this study, a low HDL in the fructose with refined wheat flour diet also lead to an increase in total cholesterol/ HDL ratio, which is considered to be an indicator of an increased risk of coronary heart disease [46].

Besides, this model showed quicker development of features of metabolic syndrome at 4 weeks. In previous studies, the period of fructose administration applied has been non-standard from 4–8 weeks to several months [23]. Previous studies have also shown that long-term fructose feeding induces mild insulin resistance without an elevation in blood pressure [47], which could be due to a protective effect of whole wheat in rat chow. Also different experimental designs for fructose administration in rats have shown to induce variable physiological responses [23].

## Conclusion

This study highlights an important finding and proposes a rat model of metabolic syndrome in 4 weeks with 60% fructose in diet replacing whole wheat flour with refined flour as a major constituent. This animal model may better translate MS in humans associated with hypertension, endothelial dysfunction, hyper-riglyceridemia, insulin resistance and a low HDL, thus offering a better model of MS. Moreover, this model also allows studying, therapeutic effects of test substances on dyslipidemia including a low HDL level. Further studies are required to study the pathological changes and to assess the extended effects of different dietary constituents on MS.

## Methods

Handling and use of animals was strictly in accordance with the Office of Laboratory Animal Welfare policy [48] and the protocol used in this study was part of PhD proposal of Ms Farida Amin, which was approved by the Aga Khan University in an open defense held on March 09, 2012. Male Sprague–Dawley rats weighing 150–200 gms, housed at the Aga Khan University animal house, Karachi and maintained at 23-25°C were given diet and tap water *ad libitum*. The following four groups were made (6 rats/ group): controls on regular diet with whole wheat flour, controls on regular diet with refined (white) wheat flour, 60% fructose in diet with whole wheat flour and 60% fructose in diet with refined wheat flour.

The diets of 2 groups which were not fed fructose contained 60% whole wheat flour or refined wheat flour. Other constituents were 20% proteins, 15% fats, salt, vitamins, minerals and potassium metabiphosphate. The diet of the fructose-fed groups contained 60% fructose in addition to the above diet mixtures after preparation.

Weight and fortnightly Blood Pressures (BP) were measured by tail cuff method as described previously [49]. Blood was drawn by tail venipuncture. Fasting serum insulin, serum fasting blood sugars (FBS), C-reactive protein (CRP), serum cholesterol (Chol), low density lipoproteins (LDL), high density lipoproteins (HDL) and triglycerides (TGL) were measured at baseline, 4 and 8 weeks of intervention. Tests were performed on Cobas c111 [50-52]. Insulin was quantified by ELISA (crystal chemicals) [53]. At the end of experiment, rats were sacrificed after anesthetizing them with ethyl ether and blood samples at 8 weeks were drawn by cardiac puncture.

To assess endothelial function, a thoracotomy was performed after adequate anesthesia, to isolate the descending thoracic aorta, which was then placed in Kreb’s solution containing (in mM) NaCl 118.2, NaHCO_3_ 25.0, CaCl_2_ 2.5, KCl 4.7, KH2PO_4_ 1.3, MgSO_4_ 1.2, glucose 11.7 (pH 7.4) aerated with a mixture of 95% oxygen and 5% CO_2_.

In a petri dish, the adherent fat and connective tissue were gently removed, and the aorta was sectioned into 2-mm segments. The instrument was calibrated and the aortic segments were mounted on hooks attached to isometric force sensors, and placed into 5 ml temperature controlled, tissue baths with Krebs’s solution, with a continuous supply of oxygen. The transducer output was continuously recorded on a computer with chart pro software and the vessels were allowed to equilibrate for 45 min. Then, the arteries were constricted with phenylephrine (PE1 μM), Sigma, St. Louis, MO and allowed to achieve maximum constriction. Acetylcholine (ACh) (Sigma) was then added in incremental log concentrations from 0.01 to 100 μM for determination of endothelium-dependent relaxation in percentage [54].

Data were analyzed on SPSS version 19.0 and Graphpad Prism version 5 and expressed as means ± SEM (Standard error of mean). One way ANOVA was used to compare the means between groups followed by Tukey’s test for multiple comparisons. Bonferonni’s test was used to compare endothelial relaxation at different doses of ACh. A P-value of < 0.05 was considered significant for a difference between groups.

## Abbreviations

MS: Metabolic syndrome; BP: Blood pressure; Chol: Cholesterol; LDL: Low density lipoprotein; HDL: High density lipoprotein; TGL: Triglycerides; CRP: C-reactive protein; FBS: Fasting blood sugars.

## Competing interests

The authors declare that they have no competing interests.

## Authors’ contributions

FA participated in developing protocol, data collection, data analysis and interpretation of data and writing manuscript. AHG contributed in conception, co-ordination, supervision and design of the study, acquisition of funding and critically revising the manuscript. Both authors read and approved the final manuscript.
